# How does capital structure change product-market competitiveness? Evidence from Chinese firms

**DOI:** 10.1371/journal.pone.0210618

**Published:** 2019-02-05

**Authors:** Li Li, Zixuan Wang

**Affiliations:** 1 The College of Finance and statistics, Hunan University, Changsha, Hunan, China; 2 Hanqing Advanced Institute of Economics and Finance, Renmin University of China, Beijing, China; University of Toronto, Rotman School, CANADA

## Abstract

Finance research shows capital structure has an important effect on the product-market competitiveness of firms. Our paper documents an asymmetric effect of capital structure on firms’ competitiveness in a sample of Chinese firms. Firms whose capital structure is characterized by a low leverage but rapid leverage growth has a dominant position in their product market. The industry average leverage ratio is also a critical factor influencing firms’ competitiveness. High debt levels hinder firms’ competitiveness. The influence of capital structure on firms’ product-market competitiveness varies based on the extent of industry concentration. In highly concentrated industries, high leverage level and slow leverage growth suppress firms’ competitiveness to a larger extent compared with industries with low concentration.

## Introduction

In recent years, studies on industrial organization and corporate finance have become increasingly interconnected. When making decisions, firms not only need to achieve the goal of value maximization but also pay close attention to the behavior of their competitors to increase their product-market competitiveness. A firm can increase its product-market competitiveness by expanding the market share, differentiating products, and increasing sales channels; all of which require the firm to possess sufficient capital. Cash is considered the most liquid asset and cash holdings are closely related to the product-market competitiveness of firms [1]. A firm with sufficient cash can seize the market share from its competitors through increased research and development spending and expanding the sales network [2]. However, because of the opportunity cost, a firm typically has limited cash holdings and thus, the capital demand cannot always be met but the financing capacity of the firm is critical. A firm with strong financing capacity is more likely to occupy an advantageous position in the market. Choosing a reasonable capital structure and ensuring strong financing capacity for a firm are vital for its long-term development [3]. The development of the corporate bond market in China lags in most Western countries. An analysis of the debt ratio of nearly 4 million industrial enterprises above the designated size from 1998 to 2013 shows the sample enterprises exhibit a clear tendency of deleveraging [4]. Considering the special circumstance in China, clarifying the influence of capital structure on a firm’s competitiveness is crucial.

Studies conducted in Western countries have proposed two theories on the influence of capital structure on a firm’s product-market competitiveness. Strategic commitment theory supports that high leverage is advantageous for increasing a firm’s competitiveness. According to this theory, shareholders tend to increase debt to obtain the capital required for expanding production because they only bear limited liability. An increased leverage ratio implies a firm is committed to performing more aggressive actions in the market. The expansion of the production scale due to the increased debt enables the firm to occupy a larger market share, thereby gaining a strategic competitive advantage [5]. This theory is also supported by Maksimovic and Bolton and Scharfstein [6,7].

By comparison, deep pocket theory supports the view that low leverage is beneficial for improving competitiveness. According to this theory, relying on external financing aggravates a firm’s financial vulnerability. A high debt level restricts a firm’s future financing capacity, preventing the firm from increasing its market share, resulting in a decrease in market competitiveness. In addition, competitors with few financial constraints may adopt predatory strategies, such as a price war, to seize the profit of firms with a high debt level and even drive them out of the market thereby occupying a larger market share [8]. The deep pocket theory is supported by Opler and Titman [9]and Campello [10].

In China, studies in this field have started late and existing empirical results have diverged. Scholars who support strategic commitment theory empirically find that leverage ratio and product-market competitiveness are correlated significantly and positively [11]. Those who support deep pocket theory indicate that firms tend to adopt a low leverage level when faced with intense market competition [12].

To investigate comprehensively the influence of capital structure on the competitiveness of firms in China, 1,882 firms in 16 industries are selected in this study. These industries are divided into eight industries with a high concentration and eight industries with a low concentration. The leverage level and leverage change rate are first adopted to measure capital structure and a fixed effect model established to examine the applicability of strategic commitment and deep pocket theories in China. The empirical results show that firms that can increase their leverage rapidly also exhibit a fast increase in their sales revenue and market share. However, the high leverage level restricts their competitiveness. Therefore, firms should find a balance between leverage growth rate and leverage level and adopt a reasonable capital structure to control the speed of debt growth. The positive effect of leverage growth rate on the increase in sales revenue and the market share is weaker in industries with high concentration than in industries with a low concentration, and the restriction imposed by the leverage level on the competitiveness is stronger.

Previous studies have indicated that a firm’s competitiveness is influenced by its competitors’ selection of capital structure [13,14]. Based on the aforementioned results, this study examines further how the deviation of a firm’s capital structure from the industry average affects its product-market competitiveness. The empirical results show the overindebtedness of a firm (i.e., the debt level is higher than the industry average) is negatively related to its product-market competitiveness. Industry concentration does not affect the conclusion.

In addition to using the leverage level to measure capital structure similar in previous studies, this study innovatively introduces the concept of the leverage change rate to investigate thoroughly the influence of capital structure on product-market competitiveness from static and dynamic perspectives.

Section 2 presents a review of the literature and the proposed hypotheses. Data collection and research methods are presented in Section 3. Sections 4 and 5 delineate descriptive statistics and empirical results, respectively. The conclusion is drawn in Section 6.

## Literature review and hypotheses

Capital structure influences a firm’s and its competitors’ selections of competitive strategies in the product market and further influences the competition results [10]. However, in previous studies, theories on the relationship between the selection of capital structure and product competitiveness have diverged.

Strategic commitment theory supports that high leverage is favorable to product-market competitiveness. Concerning output competition in the product market, debts imply a firm is committed to increasing productivity, expanding the market share, and performing more aggressive actions. After paying interest, the free cash flow remaining for shareholders decreases for a firm with increased leverage. In addition, because of the limited liability effect [15], shareholders aim to increase the production output to obtain high revenue [5,16]. In an industry with low concentration and without technological barriers and where competitors generally have low debt levels, the increase in a firm’s leverage ratio is favorable for increasing output and enhancing its product-market competitiveness [17].

By contrast, deep pocket or long purse theory which was proposed by Telser [8], supports that high-leverage firms are more likely to lose their market share to low-leverage competitors. Because new-entry firms typically require a large amount of capital, they have a higher leverage ratio than other existing firms in the industry, and their financial structures are more vulnerable. Conversely, existing firms have the advantage of obtaining capital and stronger capability to bear losses and guarantee payments. Existing firms frequently adopt predatory strategies such as launching a price war or increasing output to drive the new-entry firms out of the market and obtain higher profits. Investors determine a firm’s development prospect based on its current profit. A high-leverage firm is more likely to lose their market share to low-leverage competitors and therefore has lower profits. Such information is conveyed to investors, thereby limiting the firm’s financing capacity. Because the firm has signed contracts with creditors, the firm will encounter a liquidity crisis if it is unable to pay interest regularly. The predatory behavior of low-leverage competitors causes the firm’s profit to decrease, thereby increasing the possibility that the firm will withdraw from the market because of the liquidity crisis [7]. Because the high-leverage firm is concerned with violating contracts, the firm tends to focus on its current performance and ignores its investment in the large market share, which is related adversely to its product-market competitiveness [18]. High leverage implies the firm spends a large amount of free cash flow on paying interest, leading to a decrease in future investment capacity. Moreover, the costs of external debt or equity financing are higher than internal capital (retained earnings) because of the problems of agency costs and information asymmetry. The increased leverage indicates that the firm needs to pay a higher cost for increasing output or increasing its future investment capacity, thereby losing its advantageous position in the product market. In a highly concentrated industry, high-leverage firms are more likely to lose their market share to low-leverage competitors [7]. In particular, during the economic recession, the growth rate of their sales revenue is lower than the industry average [10] because the list price set by high-leverage firms is higher than that set by low-leverage firms [18] and the occupied market share also decreases. This scenario substantially decreases the firm’s product-market competitiveness [9].

To examine the applicability of strategic commitment and deep pocket theories, this study proposes the following hypotheses:

H1a: If strategic commitment theory is valid, high-leverage firms have higher product-market competitiveness than low-leverage firms.
H1b: If the deep pocket theory is valid, low-leverage firms have higher product-market competitiveness than high-leverage firms.

Graham and Harvey [13]indicate that when making decisions on capital structure, 81% of sample firms set goals based on the leverage ratio or range. However, managers frequently receive complex information and the accurate model of the targeted capital structure is typically unknown. Even though managers can optimize firms’ capital structure, the time cost they pay is relatively high. Thus, they tend to take competitors’ capital structure as a crucial reference (Graham and Harvey, 2001; Liang, 2016)[13,14]. The relationship between capital structure and the industry average leverage ratio serves as a vital factor that influences firms’ product-market competitiveness [19]. Hernádi and Ormos [20] report that 57.5% of the chief financial officers of the sample firms consider the debt level as a critical factor determining their firms’ reasonable amount of debts. However, no clear conclusion has been drawn regarding the influence of debt level on the competitiveness of a firm when its leverage ratio is higher than the industry average. Using debts, a firm can increase its output and profits and prevent potential competitors from entering the industry [5]. However, in an industry with high concentration, they cannot enhance further their competitiveness by increasing the leverage ratio when firms in a leading position have achieved a leverage ratio similar to the industry average [21].

To investigate how the deviation of a firm’s actual leverage level from the industry average influences its product-market competitiveness, this study proposes the following hypotheses:

H2a: When the debt level is higher than the industry average, the firm has stronger product-market competitiveness.
H2b: When the debt level is lower than the industry average, the firm has stronger product-market competitiveness.

## Data, variables, and methodology

### Data

In this study, the financial data of all nonfinancial firms listed in the CSI 300 Index during 1998–2015 are collected. Moreover, the following firms are excluded: ST firms (i.e., firms receiving “special treatment” because of their abnormal financial conditions), *ST firms (i.e., ST firms that fail to comply with the rules during the “special treatment” period), and firms listed in 2015. All firms are categorized into industries according to the criteria of the China Securities Regulatory Commission. The Herfindahl–Hirschman index of each industry is calculated as an indicator for measuring the level of industry concentration. In total, 1,882 firms from 16 industries are selected. The data are collected from the CSMAR database.

### Variables

#### Product-market competitiveness

A firm’s product-market competitiveness includes its future investment capability, financial risk tolerance, and ability to withstand price wars. Indicators, such as sales volume, sales revenue, and market share can reflect a firm’s operating status and can be used to determine whether the firm possesses sufficient capital for future investment and whether it can withstand other firms’ predatory behaviors (e.g., price wars). Thus, such indicators can represent a firm’s product-market competitiveness. Previous studies have used numerous indicators to measure a firm’s product-market competitiveness (*pmc*), including Tobin’s Q ratio [22] and sales revenue growth rate [21]. Based on these studies, to reflect a firm’s competitiveness, the growth rate of the log of sales revenue (Δ*sales*) and the growth rate of the log of market share (Δ*share*) are adopted as indicators in this study.

$$\Delta sales_{it}=\text{log}(sales_{it}/sales_{i,t-1})$$

$$\Delta share_{it}=\text{log}(share_{it}/share_{i,t-1})$$

#### Capital structure

Following the methods commonly adopted in previous studies, this study used book leverage (*Blev*) and market leverage (*Mlev*) to measure the capital structure of firms.

Blev=totaldebts/totalassets

Mlev=totaldebts/(totalmarketvalue+totaldebts)

Book leverage reflects mainly a firm’s past financial status, and it can be calculated accurately and therefore easily accepted by external investors (Myers, 1977; Graham and Harvey, 2001)[15,13]. By comparison, market leverage involves the prediction of the future financial status and is thus difficult to calculate accurately (Frank and Goyal, 2003)[19].

This study also adopted leverage changes to investigate the change in firms’ competitiveness (from a dynamic perspective) when they adjust their capital structure aside from examining the effect of firms’ leverage level on their competitiveness. Few studies have explored dynamic changes in leverage and competitiveness.

$$\Delta Blev_{it}=\text{log}(Blev_{it}/Blev_{i,t-1})$$

$$\Delta Mlev_{it}=\text{log}(Mlev_{it}/Mlev_{i,t-1})$$

#### Methodology

To verify H1, this study first investigates the effect of the leverage level on a firm’s product-market competitiveness. In reference to Campello (2006)[21], six individual characteristic variables are added to the equation, namely the change rates of the log of firm size, profitability, capital expenditure, sales expense, market-to-book ratio (M/B), and Tobin’s Q ratio. The regression equation is as follows:

$$\begin{array}{ll} \Delta pmc_{it}= & lev_{i,t-1}+D\times lev_{i,t-1}+\Delta size_{i,t-1}+\Delta profitability_{i,t-1}+\Delta investment_{i,t-1}+\\ & \Delta sellexpenses_{i,t-1}+\Delta mb_{i,t-1}+Q_{i,t-1}+\varepsilon \end{array}$$ (1)

where Δ*pmc*_it_ indicates the competitiveness of firm *i* in year *t* and is represented by the growth rate of the log of sales revenue and the growth rate of the log of market share; *lev*_i,t−1_ denotes the leverage level of firm *i* in year *t−1*, which is calculated from the perspectives of book leverage and market leverage. *D* is a dummy variable. When a firm is in a high-concentration industry, *D* = 1, and otherwise *D* = 0.

The other characteristic variables are the firm size (*size*), firm profitability (*profitability*), capital expenditure (*investment*), sales expense (*sellexpenses*), M/B (*mb*), and Tobin’s Q (Q). S*ize* is derived from total assets with a natural logarithm. *Profitability* is defined as EBITDA divided by total assets. *Investment* is the ratio of capital expenditure to total assets. *Sellexpenses* is equal to the ratio of sales expenses to total assets. *Mb* means Market-To-Book ratio, which is equal to the total market value divided by equity (the total market value on the last trading day of the year is adopted). *Q* is defined as total market value divided by total assets. The change rate of the log of each variable is calculated using the same method used for calculating the growth rate of the log of sales revenue.

To verify H1 further, this study explores the effect of leverage change rate on firms’ product-market competitiveness. Using a method similar to that for investigating the effect of the leverage level, the regression equation is proposed as follows:

$$\begin{array}{ll} \Delta pmc_{it}= & \Delta lev_{i,t-1}+D\times \Delta lev_{i,t-1}+\Delta size_{i,t-1}+\Delta profitability_{i,t-1}+\Delta investment_{i,t-1}+\\ & \Delta sellexpenses_{i,t-1}+\Delta mb_{i,t-1}+Q_{i,t-1}+\varepsilon \end{array}$$ (2)

where Δ*lev*_i,t−1_ is the leverage change rate of firm *i* in year *t−1*, which is calculated from the perspectives of the change rates of book leverage and market leverage. The dependent and characteristic variables are selected and calculated using the same aforementioned method.

The adjustment of capital structure involves asymmetries (Byoun, 2008)[23]. Firms with a leverage level higher than the target leverage face more problems of information asymmetry than those with a leverage level lower than the target leverage. Thus, through the examination of the effect of asymmetric leverage adjustment on the competitiveness of firms, the following equation is proposed:

$$\begin{array}{ll} \Delta pmc_{it}= & D_{a}\times |lev_{a}|+D_{b}\times |lev_{b}|+D\times D_{a}\times |lev_{a}|+D\times D_{b}\times |lev_{b}|+\Delta size_{i,t-1}+\\ & \Delta profitability_{i,t-1}+\Delta investment_{i,t-1}+\Delta sellexpenses_{i,t-1}+\Delta mb_{i,t-1}+Q_{i,t-1}+\varepsilon \end{array}$$ (3)

where *D*_a_ is a dummy variable; when the actual leverage level of firm *i* in year *t−1* is higher than the industry average, *D*_a_ = 1; otherwise, *D*_a_ = 0. *D*_b_ is also a dummy variable; when the actual leverage level of firm *i* in year *t−1* is lower than the industry average, *D*_b_ = 1; otherwise, *D*_b_ = 0. When the debt level of firm *i* is higher than the industry average in year *t−1*, deviation of the firm’s actual capital structure from the industry average is denoted by *|lev*_a_*|* and *|lev*_b_*|* is the deviation of firm *i*’s actual capital structure from the industry average when its debt level is lower than the industry average in year *t−1*.

$$\left|lev_{a}|=|lev-lev\_ave\right|$$

$$\left|lev_{b}|=|lev-lev\_ave\right|$$

### Descriptive statistics

Table 1 defines all variables. Table 2 presents the descriptive statistics of the variables. The mean of the book leverage of Chinese firms is 43.9% and the median is 44.6%, indicating that book leverage is distributed evenly among the firms and that most firms have a leverage level lower than 50%. The mean of market leverage is 27% and the median is 22.5%, signifying that the leverage level of the Chinese firms is even lower when their growth status is considered and when their book assets are re-estimated. From the perspective of the leverage change, Chinese firms’ annual leverage is relatively stable; the mean of the book leverage change is 2.2% and the median is 1.3%. These statistics imply that during the research period, the Chinese firms were still in the cycle of increasing leverage. However, the 99th percentile of the book leverage change is 41.9%, indicating the existence of outliers. Similarly, the market leverage change is also stable and with the existence of outliers. Thus, in this study, the variables that involve outliers are winsorized to reduce the effect of outliers on the robustness of the results.

**Table 1:** Definition of variables.

*Variable*	Meaning	Definitions	Source
***Dependent variables***			
Δ*sales*	Change of sales revenue	Log (sales_it_/sales_i,t−1_)market value)	CSMAR
Δ*share*	Change of market share	Log (share_it_/share_i,t−1_)	CSMAR
***Independent variables***			
*Blev*	Book leverage	Total debts/total assets	CSMAR
Δ*Blev*	Change of book leverage	Log (Blev_it_/Blev_i,t−1_)	CSMAR
*Mlev*	Market leverage	Total debts/(total market value + total debts)	CSMAR
Δ*Mlev*	Change of market leverage	Log (Mlev_it_/Mlev_i,t−1_)	CSMAR
*D*_a_	Above-average leverage	Dummy variable which equals to 1 if leverage is above the average leverage; otherwise, the variable is 0	Byoun(2008)
*D*_b_	Below-average leverage	Dummy variable which equals to 1 if leverage is below the average leverage; otherwise, the variable is 0	Byoun(2008)
*|lev*_a_*|*	Absolute value of above-average leverage	|lev-lev_ave|	Byoun(2008)
*|lev*_b_*|*	Absolute value of below-average leverage	|lev-lev_ave|	Byoun(2008)
***Control variables***			
Δ*size*	Change of size of sales	Log (total assets_it_/total assets_i,t−1_)	CSMAR
Δ*profitability*	Change of firm profitability	Log ((EBITDA/total assets)_it_/ (EBITDA/total assets)_i,t−1_)	CSMAR
Δ*investment*	Change of capital expenditure	Log ((capital expenditure/total assets)_it_/ (capital expenditure/total assets)_i,t−1_)	CSMAR
Δ*sellexpenses*	Change of sales expense	Log ((sales expense/total assets)_it_/ (sales expense/total assets)_i,t−1_)	CSMAR
Δ*mb*	Change of Market-To-Book ratio	Log ((total market value/equity)_it_/ (total market value/equity)_i,t−1_)	CSMAR
*Q*	Tobin’s Q	Total market value/ total assets	CSMAR

Note: The CSMAR databases provide data on shares, bonds, funds, and macroeconomics in China.

Byoun (2008) can be found in the reference section.

**Table 2:** Capital structure, product competitiveness, and individual characteristic variables.

VARIABLES	Mean	SD	p1	p25	p50	p75	p99
*Blev*	43.9%	19.5%	5.0%	29.2%	44.6%	58.9%	83.3%
Δ*Blev*	2.2%	11.9%	-28.9%	-2.5%	1.3%	5.9%	41.9%
*Mlev*	27.0%	19.4%	1.3%	11.2%	22.5%	39.5%	78.2%
Δ*Mlev*	3.3%	21.8%	-50.5%	-9.1%	3.1%	15.0%	64.0%
Δ*sales*	6.0%	13.8%	-26.4%	0.0%	5.3%	11.3%	49.1%
Δ*share*	-2.0%	14.4%	-38.0%	-8.4%	-2.2%	3.7%	40.6%
Δ*size*	0.3%	0.4%	-0.5%	0.1%	0.2%	0.4%	1.8%
Δ*profitability*	-1.7%	24.9%	-76.2%	-10.3%	-0.9%	7.4%	71.7%
Δ*investment*	-2.3%	42.6%	-113.7%	-24.0%	-3.3%	18.6%	122.0%
Δ*sellexpenses*	1.1%	19.3%	-53.8%	-6.0%	0.9%	8.1%	55.7%
Δ*mb*	-0.7%	23.3%	-58.5%	-14.9%	-2.1%	13.2%	56.6%

Note: This table shows the descriptive statistics of all variables. The sample ranges from 1998 to 2015. *Blev* is defined as the total debts / total assets; Δ*Blev* is the Log (sales_it_/sales_i,t−1_). *Mlev* is the total debts/(total market value + total debts); Δ*Mlev* is the Log (share_it_/share_i,t−1_). Δ*sales* is the Log (sales_it_/sales_i,t−1_); Δ*share* is the Log (share_it_/share_i,t−1_). Detailed definitions of other variables can be found in Table 1. SD is the standard deviation of the listed values.

Regarding competitiveness, the mean growth rate of the sales revenue of the sample firms is 6.0% and the median is 5.3%, implying that most firms display increasing market competitiveness. The mean and median of the market share growth rates are −2.0% and −2.2%, respectively and the 75th percentile is 3.7%, indicating that large firms occupy a large portion of the market share and have strong market competitiveness.

Concerning individual characteristic variables, the growth rate of the firm size of the sample firms has a mean of 0.3%, a median of 0.2%, and 99th percentile of 1.8%, thereby implying that most Chinese firms have maintained their firm size at a stable level, whereas few of them are still expanding rapidly. Furthermore, the mean and median of profitability growth are −1.7% and −0.9%, respectively. These findings reveal that as the rapid development of the macroeconomy of China has decelerated gradually, Chinese firms’ profitability growth has also slowed down. However, the 99th percentile of the profitability growth rate reaches 71.7%, indicating the existence of outliers; that is, a large amount of profits are concentrated in a small number of firms. The mean and median of the growth rate of capital expenditure are 2.3% and −3.3%, respectively and those of the growth rate of sales expenses are 1.1% and 0.9%, respectively. These values and the percentiles show the existence of a significant difference in capital expenditure and sales expenses among the Chinese firms and the strategies adopted by these firms are diverse. The mean and median of the M/B growth rate are −0.7% and −2.1%, respectively, implying that the overall market expectation toward the firms is reduced.

The deep pocket theory posits that the financing capacity of a high-leverage firm is restricted, which limits the expansion of the market share. Tables 3 and 4 display the 50th percentiles of the book capital structure and market capital structure, revealing that firms with high leverage ratios have a low leverage change rate, sales revenue growth rate, and market share growth rate. The findings regarding the capital structure and market competitiveness of the Chinese firms support the deep pocket theory.

**Table 3:** Book leverage change rate and product-market competitiveness.

Blev-level	N	Δ*Blev*			Δ*sales*			Δ*share*		
		mean	p50	sd	mean	p50	sd	mean	p50	sd
1	2919	-0.38%	-0.35%	18.30%	5.83%	4.86%	15.80%	-2.02%	-2.65%	16.60%
2	3092	2.71%	1.00%	13.40%	5.81%	5.36%	14.40%	-2.24%	-2.25%	15.30%
3	3275	2.92%	1.28%	10.60%	5.89%	5.27%	13.20%	-2.22%	-2.29%	14.00%
4	3400	2.84%	1.71%	8.09%	6.20%	5.63%	12.40%	-1.90%	-1.93%	13.10%
5	3516	2.51%	1.38%	6.10%	6.40%	5.38%	13.10%	-1.74%	-2.21%	13.30%

Note: This table shows the descriptive statistics of the change of book leverage, change of sales revenue and change of market share at different levels of book leverage. From 1 to 5, the level of book leverage is increasing. Refer to Table 1 for definitions of all variables.

**Table 4:** Market leverage change rate and product-market competitiveness.

Mlev-level	N	Δ*Mlev*			Δ*sales*			Δ*share*		
		mean	p50	sd	mean	p50	sd	mean	p50	sd
1	2,856	-1.48%	-0.91%	27.80%	6.90%	5.68%	17.20%	-0.91%	-1.75%	17.80%
2	3,138	2.89%	2.63%	24.20%	6.27%	5.70%	14.20%	-1.75%	-1.81%	15.00%
3	3,285	4.34%	3.74%	21.90%	6.00%	5.42%	13.20%	-2.13%	-2.19%	14.10%
4	3,416	5.03%	4.32%	19.00%	5.84%	5.17%	12.00%	-2.28%	-2.28%	12.60%
5	3,507	4.68%	3.47%	15.10%	5.37%	4.71%	12.20%	-2.81%	-2.86%	12.70%

Note: This table shows the descriptive statistics of the change of market leverage, change of sales revenue and change of market share at different levels of market leverage. From 1 to 5, the level of market leverage is increasing. Refer to Table 1 for definitions of all variables.

## Empirical results

Table 5 presents the effects of the leverage level on changes in sales revenues and market share.

**Table 5:** Effect of leverage level on product-market competitiveness.

	Δ*sales*	Δ*share*	Δ*sales*	Δ*share*	Δ*sale*	Δ*share*	Δ*sale*	Δ*share*
	(1)	(2)	(3)	(4)	(5)	(6)	(7)	(8)
*Blev*	-0.051***	-0.026***	-0.046***	-0.015**				
	(-4.68)	(-4.29)	(-4.88)	(-1.99)				
D*Blev			-0.070*	-0.034***				
			(-1.81)	(-3.98)				
*Mlev*					-0.068***	-0.013*	-0.069***	-0.016**
					(-5.70)	(-1.67)	(-6.74)	(-2.51)
D*Mlev							-0.203***	-0.046***
							(-3.08)	(-3.87)
Δ*size*	6.335***	7.131***	6.345***	6.164***	6.072***	6.689***	6.113***	5.974***
	(17.69)	(21.20)	(20.36)	(12.19)	(17.32)	(23.61)	(20.04)	(12.22)
Δ*profitability*	-0.019***	-0.007	-0.019***	-0.015**	-0.020***	-0.009*	-0.020***	0.002
	(-3.36)	(-1.27)	(-3.80)	(-2.40)	(-3.55)	(-1.82)	(-3.98)	(0.25)
Δ*investment*	-0.002	0.000	-0.002	-0.001	-0.003	0.000	-0.003	-0.001
	(-0.94)	(0.06)	(-1.02)	(-0.32)	(-1.12)	(0.07)	(-1.19)	(-0.25)
Δ*sellexpenses*	0.019**	0.020**	0.018***	-0.012	0.017**	0.018***	0.017**	-0.011
	(2.19)	(2.39)	(2.60)	(-1.10)	(2.04)	(2.64)	(2.42)	(-1.08)
Δ*mb*	0.044***	-0.014***	0.044***	-0.011**	0.038***	-0.019***	0.039***	-0.014***
	(9.90)	(-3.29)	(10.17)	(-2.01)	(9.16)	(-4.72)	(9.38)	(-2.71)
*Q*	0.004***	0.003***	0.004***	0.005***	-0.000	0.004***	-0.001	0.006***
	(3.01)	(2.77)	(3.43)	(3.63)	(-0.08)	(3.06)	(-0.78)	(3.65)
Constant	0.055***	-0.034***	0.057***	-0.039***	0.059***	-0.042***	0.065***	-0.048***
	(9.38)	(-8.77)	(10.82)	(-8.03)	(10.59)	(-10.81)	(12.28)	(-9.82)
Year-fixed effects	Y	Y	Y	Y	Y	Y	Y	Y
Firm-fixed effects	Y	Y	Y	Y	Y	Y	Y	Y
*Nobs*	13,455	13,455	13,455	13,455	13,455	13,455	13,455	13,455
*R*^*2*^	0.315	0.266	0.362	0.274	0.324	0.298	0.330	0.284

Note: This table presents the regression results for the relationship between leverage level and firms’ product-market competitiveness. HHI index is used to measure the industry concentration. We use both book leverage (Blev) and market leverage (Mlev) to measure the capital structure of firms. The notation “Y” (“N”) for year- and industry-fixed effects indicates that these variables are (not) controlled. Refer to Table 1 for definitions of variables. The standard errors are adjusted for potential heteroskedasticity, and the absolute values of t statistics are shown in parentheses. Significance at the 10%, 5%, and 1% levels is indicated by *, **, and ***, respectively.

According to the first and second column, the coefficients of *Blev* are −0.051 (*t* = −4.68) and −0.026 (*t* = −4.29), respectively. According to the fifth and sixth column, the coefficients of *Mlev* are −0.068 (*t* = −5.70) and −0.013 (*t* = −1.67), respectively. These findings reveal a capital structure with high leverage inhibits the growth of sales revenues and the market share, thereby reducing the product-market competitiveness of firms. In third, fourth, seventh and eighth columns, the coefficients of D*Blev and D*Mlev are negative, which reveal that when leverage is increased to the same level, the sales revenues and market share in industries with a high concentration decrease more rapidly than those in industries with a low concentration. When leverage is decreased to the same level, the competitiveness of firms in industries with a high concentration increases more rapidly than that in industries with low concentration. This finding implies that the increased leverage level suppresses firms’ product-market competitiveness more significantly in an industry with a high concentration than in an industry with a low concentration. Similarly, the decreased leverage level enhances firms’ competitiveness more significantly in an industry with high concentration than in an industry with a low concentration.

The empirical results in Table 5 show the high leverage level suppresses the rapid growth of a firm’s sales revenues and market share in the product market, and this effect is more prominent in industries with a high concentration. This result is consistent with that of Zhu et al. (2002), thereby supporting the deep pocket theory. If a firm has a capital structure with high leverage, its product-market competitiveness may be suppressed. With a continuous increase in the leverage level, the amount of debt approaches debt capacity, causing the firm’s financing capacity to decrease and financing costs to increase. Therefore, the firm has limited ability to adjust prices flexibly and loses its market share more easily to low-leverage competitors. In addition, the firm’s investments for market share expansion are limited because a large amount of free cash flow is used to pay interest. This specific scenario eventually causes its product-market competitiveness to decrease. Firms that can increase their leverage level rapidly typically have a relatively low absolute level of leverage. When leverage has reached a high level and cannot be increased further, the positive effect of the leverage increase on firm competitiveness cannot be realized.

Table 6 shows the effects of the leverage change on sales revenues and market share changes.

**Table 6:** Effect of leverage change on product-market competitiveness.

	Δ*sales*	Δ*share*	Δ*sales*	Δ*share*	Δ*sale*	Δ*share*	Δ*sale*	Δ*share*
	(1)	(2)	(3)	(4)	(5)	(6)	(7)	(8)
Δ*Blev*	0.142***	0.115***	0.147***	0.121***				
	(12.64)	(9.96)	(12.62)	(10.20)				
D*Δ*Blev*			-0.016***	-0.016***				
			(-2.80)	(-2.82)				
Δ*Mlev*					0.083***	0.071***	0.085***	0.076***
					(10.67)	(9.03)	(11.15)	(9.85)
D*Δ*Mlev*							-0.017***	-0.036***
							(-3.25)	(-3.44)
Δ*size*	4.264***	2.965***	4.284***	2.990***	4.625***	3.195***	4.636***	3.221***
	(11.72)	(8.01)	(11.77)	(8.10)	(12.85)	(8.70)	(14.18)	(9.76)
Δ*profitability*	-0.016***	-0.016***	-0.016***	-0.016***	-0.017***	-0.016***	-0.015	-0.016***
	(-2.77)	(-2.78)	(-2.79)	(-2.85)	(-2.92)	(-2.86)	(-1.43)	(-3.06)
Δ*investment*	-0.003	-0.001	-0.003	-0.001	-0.003	-0.001	-0.003	-0.001
	(-1.37)	(-0.39)	(-1.37)	(-0.38)	(-1.32)	(-0.37)	(-1.40)	(-0.41)
Δ*sellexpenses*	0.010	-0.017*	0.011	-0.017*	0.012	-0.016*	0.012*	-0.016**
	(1.24)	(-1.96)	(1.25)	(-1.95)	(1.44)	(-1.83)	(1.70)	(-2.28)
Δ*mb*	0.030***	-0.021***	0.030***	-0.021***	0.090***	0.030***	0.090***	0.030***
	(7.32)	(-4.83)	(7.36)	(-4.80)	(13.78)	(4.48)	(14.26)	(4.67)
*Q*	0.009***	0.002	0.009***	0.002	0.009***	0.002	0.009***	0.002*
	(7.44)	(1.23)	(7.44)	(1.22)	(7.60)	(1.47)	(8.60)	(1.66)
Constant	0.028***	-0.035***	0.028***	-0.035***	0.027***	-0.036***	0.027***	-0.036***
	(12.73)	(-15.15)	(12.88)	(-14.64)	(12.24)	(-15.40)	(13.17)	(-17.38)
Year-fixed effects	Y	Y	Y	Y	Y	Y	Y	Y
Firm-fixed effects	Y	Y	Y	Y	Y	Y	Y	Y
*Nobs*	13,455	13,455	13,455	13,455	13,455	13,455	13,455	13,455
*R*^*2*^	0.299	0.250	0.276	0.231	0.324	0.288	0.308	0.245

Note: This table presents the regression results for the relationship between the change of leverage and firms’ product-market competitiveness. HHI index is used to measure the industry concentration. We use both change of book leverage (ΔBlev) and change of market leverage (ΔMlev) to measure the change of capital structure of firms. The notation “Y” (“N”) for year- and industry-fixed effects indicates that these variables are (not) controlled. Refer to Table 1 for definitions of variables. The standard errors are adjusted for potential heteroskedasticity, and the absolute values of t statistics are shown in parentheses. Significance at the 10%, 5%, and 1% levels is indicated by *, **, and ***, respectively.

The coefficients of *ΔBlev* are 0.142 (*t* = 12.64) and 0.115 (*t* = 9.96), respectively (first and second columns). The coefficients of *ΔMlev* are 0.083 (*t* = 10.67) and 0.071 (*t* = 9.03), respectively (fifth and sixth columns). These findings imply that firms with a capital structure that enables rapid increases in leverage exhibit rapid growth in sales revenue and market share, thereby enhancing their product-market competitiveness. In third, fourth, seventh, and eighth columns, the coefficients of D*ΔBlev and D*ΔMlev are negative, which reveal that when leverage is increased by the same percentage, growth rates of sales revenue, and market share in industries with a low concentration are higher than those in industries with a high concentration. When leverage is decreased by the same percentage, firms’ competitiveness decreases to a larger extent in industries with low concentration than in industries with high concentration. This finding implies that compared with industries with low concentration, the rapid increase in leverage enhances firms’ product-market competitiveness to a lesser extent in highly concentrated industries, and the rapid decrease in leverage suppresses firms’ competitiveness to a lesser extent in highly concentrated industries.

The empirical results displayed in Table 6 reveal that the rapid increase in leverage facilitates the rapid growth of firms’ sales revenue and market share in the product market. This effect is more significant in industries with a low concentration. This result is consistent with that of Liu et al. (2003), supporting strategic commitment theory. Thus, debt can help firms to strengthen their product-market competitiveness. Firms that can increase leverage rapidly have strong ability to obtain capital sufficient to enable the firms to expand their size, increase investment, acquire competing firms, and elevate their position in the market. In addition, the need to pay interest prompts managers to exert more effort into maximizing the value of their firms, which improves firms’ performance and enhances their product-market competitiveness.

Table 7 presents the effects of the deviation of a firm’s leverage level from the industry average on sales revenues and market share changes.

**Table 7:** Effect of leverage deviation from the industry average on product-market competitiveness.

	Δ*sales*	Δ*share*	Δ*sales*	Δ*share*	Δ*sales*	Δ*share*	Δ*sales*	Δ*share*
	(1)	(2)	(3)	(4)	(5)	(6)	(7)	(8)
*D*_a_*|*Blev*_a_*|*	-0.097***	0.038***	-0.099***	0.028***				
	(-6.60)	(2.72)	(-6.45)	(3.06)				
*D*_b_*|*Blev*_b_*|*	0.051***	0.046***	0.047***	0.080***				
	(3.04)	(2.61)	(2.65)	(5.98)				
D*D_a_*|*Blev*_a_*|*			0.019	-0.016				
			(0.38)	(-0.70)				
D*D_b_*|*Blev*_b_*|*			0.048	0.057				
			(0.80)	(1.40)				
*D*_am_*|*Mlev*_am_*|*					-0.133***	0.135***	-0.133***	0.104***
					(-10.22)	(3.91)	(-9.82)	(2.97)
*D*_bm_*|*Mlev*_bm_*|*					0.075***	0.328**	0.067***	0.191**
					(4.05)	(2.21)	(3.49)	(2.16)
D*D_am_*|*Mlev*_am_*|*							-0.004	-0.020
							(-0.09)	(-1.11)
D*D_bm_*|*Mlev*_bm_*|*							0.084	-0.001
							(1.31)	(-0.07)
Δ*size*	6.356***	4.382***	6.357***	6.913***	6.131***	4.031***	6.130***	4.029***
	(18.06)	(12.31)	(18.06)	(21.05)	(17.56)	(7.70)	(17.56)	(7.72)
Δ*profitability*	-0.020***	-0.021***	-0.020***	-0.009*	-0.023***	-0.022***	-0.023***	-0.022***
	(-3.49)	(-3.62)	(-3.49)	(-1.71)	(-4.04)	(-3.25)	(-4.06)	(-3.31)
Δ*investment*	-0.003	0.000	-0.003	0.000	-0.004	-0.002	-0.004	-0.002
	(-1.09)	(0.08)	(-1.09)	(0.08)	(-1.49)	(-0.51)	(-1.49)	(-0.55)
Δ*sellexpenses*	0.017**	-0.010	0.017**	0.019**	0.016*	-0.033***	0.016*	-0.032***
	(2.07)	(-1.14)	(2.07)	(2.32)	(1.88)	(-3.01)	(1.88)	(-2.99)
Δ*mb*	0.043***	-0.016***	0.043***	-0.016***	0.042***	-0.012**	0.042***	-0.012**
	(10.18)	(-3.62)	(10.18)	(-3.84)	(9.95)	(-2.14)	(9.96)	(-2.14)
*Q*	0.004***	0.000	0.004***	0.003***	0.000	0.001	0.000	0.001
	(2.92)	(0.20)	(2.93)	(3.45)	(0.26)	(0.58)	(0.26)	(0.68)
Constant	0.039***	-0.040***	0.039***	-0.052***	0.045***	-0.043***	0.045***	-0.044***
	(13.19)	(-13.52)	(13.19)	(-23.74)	(15.21)	(-9.13)	(15.22)	(-9.24)
Year-fixed effects	Y	Y	Y	Y	Y	Y	Y	Y
Firm-fixed effects	Y	Y	Y	Y	Y	Y	Y	Y
*Nobs*	13,455	13,455	13,455	13,455	13,455	13,455	13,455	13,455
*R*^*2*^	0.276	0.243	0.265	0.231	0.302	0.293	0.291	0.288

Note: This table presents the regression results for the relationship between the deviation of leverage from the industry average and firms’ product-market competitiveness. HHI index is used to measure the industry concentration. We use both deviation of book leverage (|Blev|) and deviation of market leverage (|Mlev|) to measure the deviation of capital structure from industry average. The notation “Y” (“N”) for year- and industry-fixed effects indicates these variables are (not) controlled. Refer to Table 1 for definitions of variables. The standard errors are adjusted for potential heteroskedasticity, and the absolute values of t statistics are shown in parentheses. Significance at the 10%, 5%, and 1% levels is indicated by *, **, and ***, respectively.

According to the first and second columns in Table 7, when a firm’s leverage level is higher than the industry average, the coefficients of *D*_a_*|*Blev*_a_*|* are −0.097 (*t* = −6.60) and 0.038 (*t* = 2.72), respectively. When the leverage level is lower than the industry average, the coefficients of *D*_b_*|*Blev*_b_*|* are 0.051 (*t* = 3.04) and 0.046 (*t* = 2.61), respectively. The results for market leverage are the same as those for market leverage (as shown in the fifth and sixth columns).

We also find the high and low industry concentrations have no significant difference when the leverage of firms deviates from the average of industry leverage. The coefficients of D*D_a_*|*Blev*_a_*|* and D*D_b_*|*Blev*_b_*|* found in the third and fourth columns are not significant. In addition, we detect the same empirical results in the market leverage found in the seventh and eighth columns.

The results indicate that when firms’ leverage level is lower than the industry’s average, continuously increasing their leverage is favorable for the growth of sales revenues. The firms can raise sufficient capital through debts, which ensures their future financing capacity and is beneficial for strengthening their product-market competitiveness. By comparison, when firms’ leverage level is higher than the industry average, their sales revenue growth is suppressed. Overindebtedness causes firms to have high-interest costs and restricts their ability to adjust prices flexibly. Therefore, these firms can easily lose their market share to competitors. Under the effect of industry factors, product-market competitiveness eventually decreases as the degree of deviation of firms’ leverage level from the industry average increases. Although market share increases regardless of whether the leverage level is higher or lower than the industry average among firms with the same degree of deviation, firms with lower leverage level than the industry average exhibit a more rapid growth of the market share.

## Conclusion

Capital structure affects firms’ product-market competitiveness from the aspects of leverage level and leverage change. The rapid increase in leverage facilitates firms to possess sufficient capital, which is favorable for the expansion of their firm size, increase in investments and helping them occupy a dominant position in the market. However, the increase in the leverage level leads to an increase in interest costs and a decline in future financing capacity, which is disadvantageous for the continuous increase in firms’ competitiveness. Thus, firms should find a balance between high and low levels of leverage. Leverage should be increased reasonably while avoiding the occurrence of a liquidity crisis. Compared with industries with low concentration, leverage growth enhances firms’ competitiveness to a lower extent in highly concentrated industries; whereas leverage level suppresses firms’ competitiveness to a larger extent in highly concentrated industries. Therefore, firms should control their debt at a low level. The industry average leverage ratio affects firms’ competitiveness. High-leverage firms are likely to lose their market share to low-leverage competitors. Thus, the firm should maintain its debt level below the industry average.

This study makes three major contributions. First, this study analyzes the influence of capital structure on the product-market competitiveness of listed firms in China and extends empirical research on the financial and market strategies of Chinese firms. Second, this study finds that strategic commitment and deep pocket theories are not contradictory, but they are compatible with each other. They can be regarded as different market strategies under different financial structures of firms. Finally, this study investigates the capital structure and market competitiveness from the perspective of the market structure, thereby expanding the understanding of firms’ operational strategies.
